# Exercise Training Prevents Oxidative Stress and Ubiquitin-Proteasome System Overactivity and Reverse Skeletal Muscle Atrophy in Heart Failure

**DOI:** 10.1371/journal.pone.0041701

**Published:** 2012-08-03

**Authors:** Telma F. Cunha, Aline V. N. Bacurau, Jose B. N. Moreira, Nathalie A. Paixão, Juliane C. Campos, Julio C. B. Ferreira, Marcelo L. Leal, Carlos E. Negrão, Anselmo S. Moriscot, Ulrik Wisløff, Patricia C. Brum

**Affiliations:** 1 School of Physical Education and Sport, University of São Paulo, São Paulo, Brazil; 2 Biomedical Sciences Institute, University of São Paulo, São Paulo, Brazil; 3 Heart Institute (InCor), University of São Paulo, São Paulo, Brazil; 4 K. G. Jebsen Center of Exercise in Medicine, Norwegian University of Science and Technology, Trondheim, Norway; University of Rome La Sapienza, Italy

## Abstract

**Background:**

Heart failure (HF) is known to lead to skeletal muscle atrophy and dysfunction. However, intracellular mechanisms underlying HF-induced myopathy are not fully understood. We hypothesized that HF would increase oxidative stress and ubiquitin-proteasome system (UPS) activation in skeletal muscle of sympathetic hyperactivity mouse model. We also tested the hypothesis that aerobic exercise training (AET) would reestablish UPS activation in mice and human HF.

**Methods/Principal Findings:**

Time-course evaluation of plantaris muscle cross-sectional area, lipid hydroperoxidation, protein carbonylation and chymotrypsin-like proteasome activity was performed in a mouse model of sympathetic hyperactivity-induced HF. At the 7^th^ month of age, HF mice displayed skeletal muscle atrophy, increased oxidative stress and UPS overactivation. Moderate-intensity AET restored lipid hydroperoxides and carbonylated protein levels paralleled by reduced E3 ligases mRNA levels, and reestablished chymotrypsin-like proteasome activity and plantaris trophicity. In human HF (patients randomized to sedentary or moderate-intensity AET protocol), skeletal muscle chymotrypsin-like proteasome activity was also increased and AET restored it to healthy control subjects’ levels.

**Conclusions:**

Collectively, our data provide evidence that AET effectively counteracts redox imbalance and UPS overactivation, preventing skeletal myopathy and exercise intolerance in sympathetic hyperactivity-induced HF in mice. Of particular interest, AET attenuates skeletal muscle proteasome activity paralleled by improved aerobic capacity in HF patients, which is not achieved by drug treatment itself. Altogether these findings strengthen the clinical relevance of AET in the treatment of HF.

## Introduction

HF is a syndrome of poor prognosis characterized by exercise intolerance, early fatigue and skeletal myopathy marked by atrophy and shift toward fast twitch fibers [1], [2], which may culminate in cardiac cachexia, an underestimated problem for HF prognosis and healthcare expenditure [3]. Pathophysiological determinants of skeletal myopathy in HF have begun to be elucidated and a dynamic imbalance of anabolic and catabolic processes has been proposed [4]. In fact, increased protein degradation, circulating proinflammatory cytokines and oxidative stress are common features of systemic diseases-induced skeletal muscle wasting, including HF [5]–[8].

UPS is a major proteolytic pathway responsible for disposal of damaged proteins, which accumulate in skeletal myopathies [9]. Indeed, aggravation of skeletal muscle atrophy is associated with UPS overactivation [9]. Atrogin-1 and MuRF1, E3 ligases driving conjugation of ubiquitin chains to proteasome substrates, are not only directly associated with but required for skeletal muscle atrophy [10], [11], highlighting the importance of UPS beyond associative findings. Despite the important role played by UPS in atrophying states, little is known about its involvement in HF-induced muscle atrophy.

The mechanisms underlying UPS overactivation in skeletal myopathies have not been clarified. However, attention should be driven to oxidative stress due to its differential modulation UPS activation [12], [13]. Even mild disturbance of redox balance causes protein oxidation, leading to proteasomal overactivation for maintenance of cell viability [14]. Furthermore, increased oxidative stress in HF has been associated with early fatigue and skeletal myopathy [15], [16]. However, the association among oxidative stress, UPS activation and skeletal muscle atrophy in HF has been poorly addressed.

Even though muscle wasting is considered an independent predictor of mortality in human HF [17], no available medication is effective in counteracting HF skeletal myopathy. Therefore, alternative therapies are of clinical relevance. AET has been established as an adjuvant therapy for HF, counteracting exercise intolerance and improving quality of life [18], [19]. Additionally, studies demonstrate beneficial effects of AET on skeletal muscle structure and function in chronic diseases [20], [21], however, its impact on skeletal muscle UPS activation remains poorly understood.

Using a mice model of sympathetic hyperactivity-induced HF through disruption of α_2A_ and α_2C_ adrenergic receptor genes (*α_2A_/α_2C_ARKO* mice) [22], [23], we hypothesized that: (a) UPS would be up-regulated in plantaris muscle of *α_2A_/α_2C_ARKO* mice and associated with increased oxidative stress and muscle atrophy; (b) AET would counteract HF-induced skeletal muscle oxidative damage, UPS overactivation and atrophy. In addition, using vastus lateralis muscle biopsies from HF patients and age-matched healthy individuals, we tested the hypothesis that: (c) Proteasome activity would also be increased in HF patients and (d) AET would re-establish proteasome activity to healthy control levels, providing novel insights into molecular mechanisms controlling skeletal muscle phenotype in human HF and reinforcing the clinical relevance of AET as an adjuvant therapy for HF.

## Methods

### Ethical Statement

The animal care and protocols in this study were reviewed and approved by the Ethical Committee of the University of São Paulo (CEP #2007/28).

The human study was performed according to the Helsinki declaration and was approved by the Regional Committee for Medical Research Ethics in Norway (*Regionale Komiteer for Medisinsk og Helsefaglig Forskningsetikk, REK midt*) (clinical trial identifier NCT00218933). The CONSORT (Consolidated Standards of Reporting Trials) checklist for the study is available in Information S1. Written consent was obtained from all patients.

### Mice study

#### Study population

Mice with genetic disruption of both α_2A_ and α_2C_ adrenergic receptors (*α_2A_/α_2C_ARKO* mice) were used in the present study. The absence of these receptors leads to substantial increase in sympathetic tone, since they are presynaptic receptors regulating noradrenaline release in sympathetic nerve terminals [22]. Previous studies of our group demonstrated that those mice provide a physiologically relevant HF animal model [23]–[27]. Male *α_2A_/α_2C_ARKO* mice in a C57Bl6/J genetic background and their wild type controls (WT) were studied at 3, 5 or 7 months of age. Subsets of animals were allocated into time-course evaluation of skeletal muscle atrophy (WT and *α_2A_/α_2C_ARKO* at 3, 5 or 7 months of age, n = 6 per group) or evaluation of UPS activation and AET effects (7 month-old untrained WT and *α_2A_/α_2C_ARKO* [ARKO], and trained *α_2A_/α_2C_ARKO* [ARKOT], n = 6 per group). This time point was chosen because *α_2A_/α_2C_ARKO* mice display severe HF and established skeletal muscle myopathy at 7 months of age [21], [24], [28]. Additionally, α_2A_ and α_2C_ adrenoceptors have never been reported as modulators of skeletal muscle function or structure. Mice were maintained in a light (12–h light cycle) and temperature (22°C) controlled environment and with free access to standard laboratory chow (Nuvital Nutrientes, Brazil) and tap water. This study was conducted in accordance with the ethical principles in animal research adopted by the Brazilian College of Animal Experimentation (www.cobea.org.br). The animal care and protocols in this study were reviewed and approved by the Ethical Committee of the University of São Paulo (CEP #2007/28).

#### Echocardiographic assessment

Left ventricular function was assessed by M-mode echocardiography in halothane-anesthetized WT and *α_2A_/α_2C_ARKO* mice. Mice were positioned in supine position and ultrasound transmission gel was applied to the precordium. Echocardiography was performed using an Acuson Sequoia model 512 echocardiographer (Siemens, USA) equipped with a 14-MHz linear transducer. LV systolic function was estimated by fractional shortening (FS) as follows: FS (%) = [(LVEDD−LVESD)/LVEDD]×100, where LVEDD means left ventricular end-diastolic dimension, and LVESD means left ventricular end systolic dimension.

#### Exercise testing

Graded treadmill tests until exhaustion were performed as previously described by our group [29]. Running performance, here assessed by total distance run, was used to verify exercise intolerance in HF animals. Exercise tolerance was evaluated by graded treadmill running tests after adaptation to treadmill exercises over a week (10 min/day). Treadmill speed started at 6 m/min and was increased by 3 m/min every 3 minutes until exhaustion, when mice were no longer able to run. Tests were carried out by a single observer (TFC), blinded to mice’s identity. Total distance run (meters) and peak workload (m/min) were recorded.

#### Aerobic exercise training

Mice were submitted to moderate-intensity treadmill running during eight weeks (from 5 to 7 months of age), five days/week. This age was chosen due to our previous findings demonstrating substantial cardiovascular improvements by AET at a time point (7mo-old) when *α_2A_/α_2C_ARKO* mice display severe cardiac dysfunction [30], [31]. Each session consisted of 60-minute running at 60% of maximal workload achieved in a graded treadmill running test (protocol is described above), corresponding to maximal lactate steady state (MLSS), as we have previously described for the same animal model [29]. At the end of the fourth training week, animals were reevaluated for running performance in order to adjust AET intensity. Untrained mice were exposed to treadmill exercise (5 min at 40% of maximal workload, 3 days/week) in order to maintain running skills [29].

#### Skeletal muscle cross-sectional area

Forty-eight hours after the last functional assessment mice were killed and plantaris muscle was carefully harvested, snap-frozen in isopentane and stored in liquid nitrogen or −80°C depending on intended experiments. Plantaris muscle was used due to the high prevalence of type II fibers, known to display greater damage in pathological states and superior response to mechanical overload than type I fibers. Muscles were cut into 10 µm-thick sections using a cryostat (Criostat Micron HM505E, Walldorf, Germany) and incubated for myofibrillar ATPase activity after alkali (pH 10.3) preincubation. Whole muscle cross-sectional area (CSA) was evaluated at ×200 magnification and further analyzed on a digitalizing unit connected to a computer (Image Pro-plus, Media Cybernetic, USA). All analyses were conducted by a single observer (AVNB), blinded to the mice’s identity.

#### Lipid hydroperoxidation

Lipid hydroperoxides were evaluated using the ferrous oxidation-xylenol (FOX) orange technique [32]. Plantaris samples were homogenized (1∶20 wt/vol) in phosphate buffered saline (PBS; 100mM, pH 7.4) and centrifuged at 12000g for 20 min at 4°C. Pellet was discarded and supernatant was precipitated with trichloroacetic acid (10% wt/vol) and centrifuged at 12000 g for 20 min at 4°C. Supernatant was mixed with FOX reagent containing 250 mM ammonium ferrous sulfate, 100mM xylenol orange, 25 mM H_2_SO_4_, and 4 mM butylated hydroxytoluene in 90% methanol and incubated at room temperature for 30 min. Absorbance of samples was read at 560 nm.

#### Real-time PCR

Total RNA was isolated from plantaris samples using Trizol (Invitrogen, Carlsbad, California). RNA concentration and integrity were assessed. cDNA was synthesized using reverse transcriptase at 70°C for 10 min, followed by incubation at 42°C for 60 min and at 95°C for 10 min. The genes analyzed were: atrogin-1/MAFbx, MuRF-1, E3-α, USP14, USP19, USP28 and Cyclophilin (reference gene). All primers were synthesized by Invitrogen (sequences available in Information S2). Real time PCR for all genes were run separately and amplifications were performed by ABI Prism 5700 Sequence Detection System (Applied Biosystems, USA) by using SYBR Green PCR Master Mix (Applied Biosystems, USA). Results were quantified as Ct values, where Ct is defined as the threshold cycle of the polymerase chain reaction at which the amplified product is first detected. Expression was normalized by cyclofilin levels as an endogenous reference. WT group levels were arbitrarily set to 1.

#### Skeletal muscle protein expression

20S proteasome subunits (α5, α7, β1, β5, β7 subunits) (Abcam item #ab22673), polyubiquitinated proteins (Biomol item #BML-PW0930) and carbonylated protein abundance (Millipore item #S7150) were evaluated by western blotting in total extracts of plantaris muscle from WT, *α_2A_/α_2C_ARKO* and trained *α_2A_/α_2C_ARKO*. Frozen muscles were homogenized in a buffer containing 1 mM EDTA, 1 mM EGTA, 2 mM MgCl_2_, 5 mM KCl, 25 mM HEPES, pH 7.5, 100 µM PMSF, 2 mM DTT, 1% Triton X-100, and protease inhibitor cocktail (1∶100, from Sigma-Aldrich). Centrifugation was performed for 15 minutes at 10000g and 4°C, pellet was discarded and supernatant (cytosolic proteins) was used. Samples were subjected to SDS-PAGE in polyacrylamide gels (10%). Proteins were electrotransferred to nitrocellulose membrane. Equal loading of samples and transfer efficiency were monitored with the use of 0.5% Ponceau S staining of the blotted membrane. Membrane was then incubated in a blocking buffer (5% nonfat dry milk, 10 mM Tris-HCl, 150 mM NaCl, and 0.1% Tween 20, pH 7.6) for 2 h and then incubated overnight at 4°C with specific antibodies against 20S proteasome subunits (Biomol International, USA. Bands quantified in the 55-130 kDa range) and ubiquitinated proteins (Biomol International, USA. Bands quantified in the 55–130 kDa range). Protein carbonylation was assessed by measuring the levels of carbonyl groups using the OxyBlot Protein Oxidation Detection Kit (Millipore, USA), following manufacturer’s instruction. Binding of the primary antibody was detected with the use of peroxidase-conjugated secondary antibodies (rabbit or mouse, 2h) and developed using enhanced chemiluminescence detected by autoradiography. Analysis of blots was performed with Image J software (NIH, USA). Results are expressed as percentage of age-matched WT group.

#### Assay of 26 S proteasome activity

Chymotrypsin-like activity of proteasome was assayed using the fluorogenic peptide (LLVY-MCA, Enzo Life Sciences item #P802-0005). Assays were carried out in a microtiter plate by diluting 25 µg of cytosolic protein into 200 µL of 10 mM MOPS, pH 7.4 containing 25 µM LLVY-MCA (substrate), 25 µM ATP and 5.0 mM Mg^2+^. Rate of fluorescent product formation was measured with excitation and emission wavelengths of 350 and 440 nm, respectively. Peptidase activities were measured in the absence and presence (20 µM) of the proteasome-specific inhibitor epoxomicin and the difference between the two rates was attributed to the proteasome.

### Human Study

#### Patients

**Figure 1:**
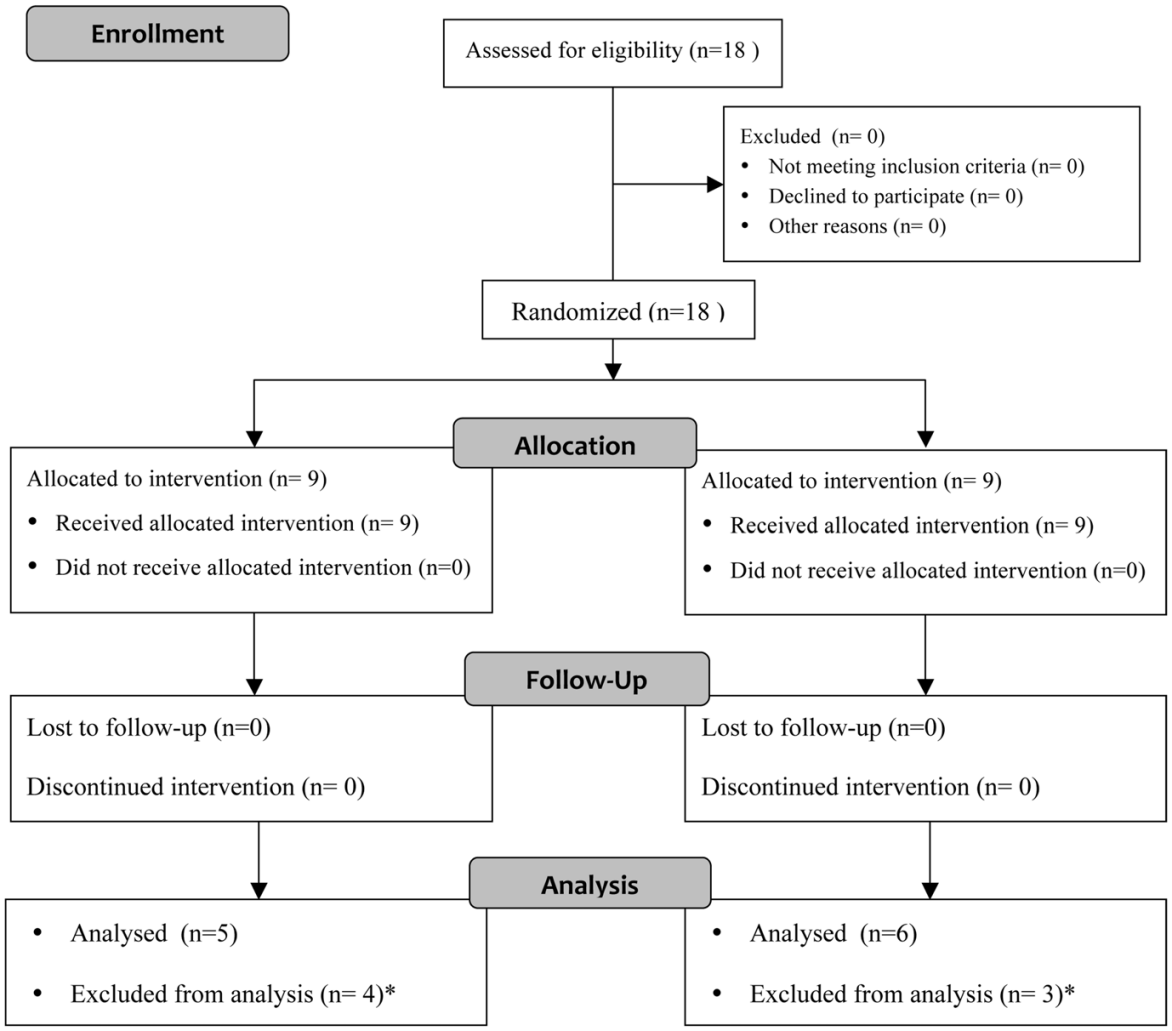
Flowchart of intervention assignment of HF patients. *Skeletal muscle biopsies were not taken from these patients, therefore, they were excluded from analysis.

Subjects were recruited from the Department of Cardiology, St. Olav’s Hospital, Trondheim, Norway, and agreed to participate in the study. The protocol started in October 2001 and ended in September 2005 due to study completion. None of the HF patients had myocardial infarction in the 12 months preceding the study. All HF patients exhibited LV ejection fraction <40% (functional class II-III, NYHA), were clinically stable and had received β-blockers, ACE inhibitors and statins for >12 months. HF patients were randomly assigned to either sedentary (HF-S) or exercise training protocol (HF-T). Subjects were randomized and stratified (by age) to HF-S or HF-T. Flowchart of patient allocation is shown in Figure 1. The randomization code was developed with a computer random-number generator to select random permuted blocks. Participants were blinded to assigned intervention. Physiological parameters such as cardiac structure and function, aerobic capacity, height and body weigh were similar between HF groups before experimental protocol. Exclusion criteria were unstable angina pectoris, uncompensated heart failure, myocardial infarction during the past 4 weeks, complex ventricular arrhythmias, no use of β-blockers and ACE inhibitors, and orthopedic or neurological limitations to exercise. Medications did not change during the 12-week study period. The study was performed according to the Helsinki declaration and was approved by the regional medical research ethics committee (clinical trial identifier NCT00218933). Written consent was obtained from all patients.

#### Exercise testing

After a 10-minute warm-up, a VO_2_peak test (MetaMax II, Cortex, Germany) was performed with an individualized treadmill ramp protocol and increased inclination by 2% when oxygen uptake stabilized at each workload until VO_2_peak was reached. Leveling off of oxygen uptake despite increased workload and respiratory exchange ratio above 1.05 were used as criteria for maximal oxygen uptake. Immediately after this workload, blood was drawn from a fingertip for measurement of lactate concentration.

#### Aerobic exercise training

HF-T group met for supervised training twice weekly and performed 1 weekly session at home. HF-S and Control groups met for supervised exercise once every three weeks. Training sessions consisted of “uphill” continuous walking at 60% of VO_2_peak (60% to 70% of peak heart rate) during 50 minutes. All subjects used a heart rate monitor (Polar Electro, Finland) to obtain the assigned exercise intensity. Borg 6-to-20 scale was used to assess the rate of perceived exertion during and after each training session. Treadmill speed and inclination were adjusted continuously to ensure that every training session was carried out at the assigned heart rate. Home-based training intensity was recorded twice by heart rate monitors, placed so that the patients were unable to see their heart rate during the exercise. Recordings confirmed correct exercise intensity during home training. Patients were instructed to immediately stop home-based training if they had chest pain or any other distressing symptoms and contact the emergency department at the hospital. The control group was told to follow advice from their family doctor with regard to physical activity. In addition, they met for 47 minutes of continuous treadmill walking at 70% of peak heart rate every 3 weeks.

#### Skeletal muscle biopsies

Skeletal muscle biopsy samples were obtained from the vastus lateralis with a sterile 5 mm-diameter biopsy needle (Bergstrom) under local anesthesia. Sample preparation, proteasome activity and protein ubiquitination and carbonylation measurements were carried out as described above for mice samples.

### Statistical Analysis

All values are presented as means ± standard error from mean. Data were tested for normal distribution and one-way analysis of variance (ANOVA) followed by Duncan post hoc testing was used to compare all variables. Statistical significance was considered achieved when P value was set as <0.05.

## Results

### Physiological Parameters in α2A/α2CARKO Mice

Corroborating our previous findings [21], [26], [30], Table 1 shows cardiac function deterioration in α_2A_/α_2C_ARKO mice from 3 to 7 months of age, latter associated with signs of HF as cardiac enlargement and severe dysfunction, lung edema and exercise intolerance. It was also shown by our group that α_2A_/α_2C_ARKO mice display severe pathological cardiac remodeling, activation of renin-angiotensin system and impaired calcium handling [24], [27], [28], [33], which are paralleled by a 30% mortality rate observed at the 7^th^ month of age (Information S3), supporting the rationale of using α_2A_/α_2C_ARKO mice as a model of severe HF.

**Table 1:** α_2A_/α_2C_ARKO mice physiological parameters.

	3 month-old		5 month-old		7 month-old	
	WT	ARKO	WT	ARKO	WT	ARKO
**Body mass (g)**	26±0.5	25±0.4	27±0.5	29±0.3	30±0.3	28±0.3
**FS, %**	21±0.5	17±0.2	22±0.4	15±0.3*	22±0.7	15±0.5*
**LVEDD (mm)**	0.38±0.01	0.38±0.01	0.39±0.01	0.40±0.001	0.38±0.01	0.41±0.01*
**Distance run (m)**	403±28	362±19	397±10	331±13	355±18	235±21*

Body mass, left ventricular fractional shortening (FS), left ventricular end-diastolic diameter (LVEDD) and running performance in 3, 5 and 7 month-old wild type (WT) and α_2A_/α_2C_ARKO mice. Data were analyzed by one-way ANOVA followed by Duncan post hoc test and are presented as mean ± standard error from mean. *p<0.05 vs. age-matched WT.

### Time-course of Skeletal Muscle Trophicity, Oxidative Stress and Proteasome Activation in α2A/α2CARKO Mice

**Figure 2:**
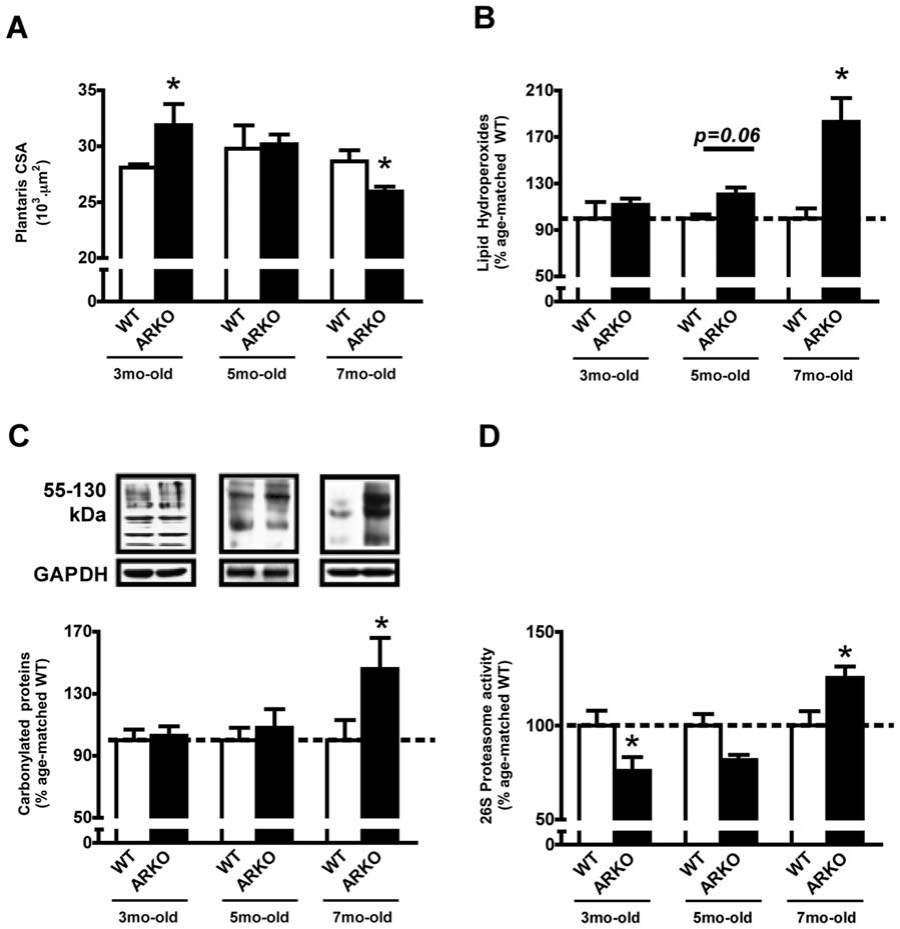
Plantaris trophicity, oxidative stress and chymotrypsin-like proteasomal activity in α_2A_/α_2C_ARKO mice. Plantaris muscle cross-sectional area (CSA) (A), lipid hydroperoxidation (B), protein carbonylation (C) and chymotrypsin-like proteasome activity (D) in 3, 5 and 7 month-old wild type (WT) and α_2A_/α_2C_ARKO mice (ARKO). Immunobloting data are shown as percentage of age-matched WT group (set to 100%)). Representative images of immunoblots are shown below respective charts. Data are presented as mean ± standard error from mean. *p<0.05 vs. age-matched WT.

Severity of cardiac dysfunction was paralleled by progressive reduction of plantaris muscle CSA from 3 to 7 months of age in α_2A_/α_2C_ARKO mice in comparison with age-matched WT (Figure 2A). Plantaris muscle hypertrophy was observed in α_2A_/α_2C_ARKO mice at 3 months of age, which was no longer observed at the 5^th^ month, when α_2A_/α_2C_ARKO plantaris CSA was similar to age-matched WT mice. Finally, 7 month-old α_2A_/α_2C_ARKO mice displayed plantaris muscle atrophy. Representative histological images are shown in Information S4. Lipid hydroperoxidation in plantaris muscle were similar between groups at the 3^rd^ month, progressed to a strong trend toward elevation in 5 month-old α_2A_/α_2C_ARKO mice and culminated in significantly increased lipid hydroperoxidation in 7 mo-old α_2A_/α_2C_ARKO mice when compared with age-matched WT (Figure 2B). Likewise, protein carbonylation was elevated only in 7 mo-old α_2A_/α_2C_ARKO mice, when HF was present (Figure 2C). Skeletal muscle proteasomal activity was significantly decreased in 3 mo-old α_2A_/α_2C_ARKO mice, progressing to unchanged levels at the 5^th^ month, ultimately reaching significant elevation in 7 mo-old α_2A_/α_2C_ARKO mice compared with age-matched WT (Figure 2D). As plantaris atrophy and oxidative damage were observed only at 7 months of age, further experiments were carried out at this time point.

### Effects of AET on Cardiac Function and Lung Water Content in α2A/α2CARKO Mice

As shown in Table 2, AET restored FS of α_2A_/α_2C_ARKO to WT’s values. Lung edema was also prevented by AET in α_2A_/α_2C_ARKO mice (Table 2), which corroborates our previous findings addressing cardiac function in this model [26], [30], [33]. Improved cardiac function after AET was associated with reduced mortality rate (Information S3) at the 7^th^ month of age.

**Table 2:** Cardiac function and lung water content.

	WT	ARKO	ARKOT
**FS, %**	24±1	14±1*	19±1
**Lung wet/dry ratio**	5,57±0,13	6,58±0,49*	5,82±0,22#

Left ventricular fractional shortening (FS) and lung wet/dry ratio in 7 month-old wild type (WT), untrained (ARKO) and trained α_2A_/α_2C_ARKO mice ARKOT. Data are presented as mean ± standard error from mean.

* p<0.05 vs. age-matched WT.

# p<0,05 vs. age-matched ARKO.

### mRNA Levels of Skeletal Muscle UPS Components in α2A/α2CARKO Mice and Effects of AET

**Figure 3:**
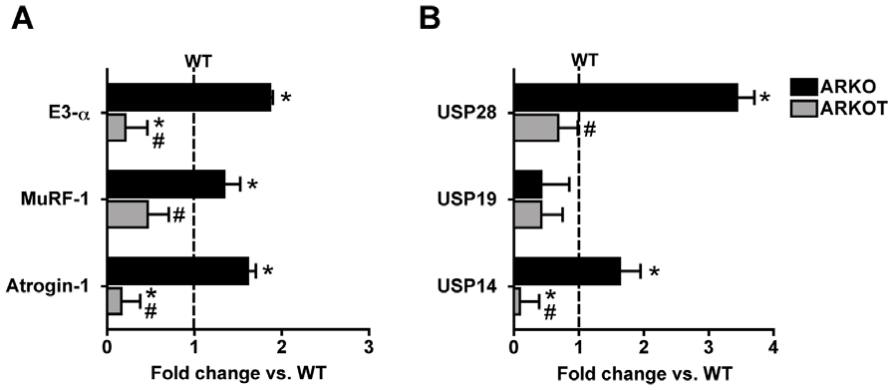
mRNA levels of UPS components. mRNA levels of E3 ligases E3-α, MuRF-1 and Atrogin-1 (A), and deubiquitinating enzymes USP28, USP19 and USP14 (B). WT values were arbitrarily set to 1.0 and are represented by the dashed line. Data are shown as fold change over wild type control group and presented as mean ± standard error from mean. *p<0.05 vs. WT; #p<0.05 vs. age-matched ARKO.

To further investigate the contribution of UPS components for plantaris atrophy, we evaluated E3 ligases mRNA expression in 7 mo-old α_2A_/α_2C_ARKO mice (Figure 3A). α_2A_/α_2C_ARKO mice displayed higher Atrogin-1/MAFbx and E3α mRNA levels than age-matched WT (dashed line), which were effectively reduced by AET (Figure 3A). Deubiquitinating enzymes may also modulate protein tagging for proteasomal degradation, therefore, we evaluated USP28, USP19 and USP14 mRNA levels. Figure 3B shows that HF mice displayed increased USP28 and 14 mRNA levels. USP28 mRNA levels were reestablished to WT levels while USP14 mRNA levels were reduced to values below WT’s (Figure 3B).

### Skeletal Muscle UPS Activation in α2A/α2CARKO Mice and Effects of AET

**Figure 4:**
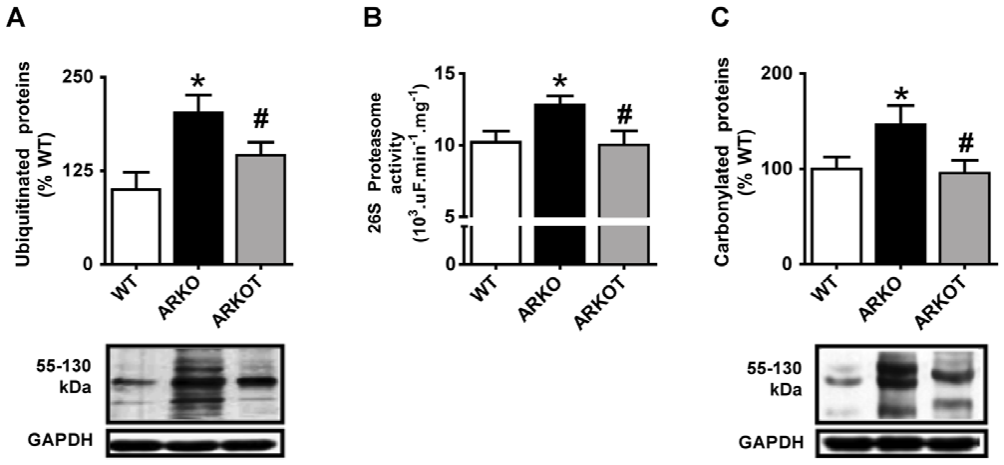
UPS activation and effects of AET. Protein ubiquitination (A), chymotrypsin-like proteasome activity (B) and protein carbonylation (C) in 7 month-old wild type (WT), untrained α_2A_/α_2C_ARKO (ARKO) and trained α_2A_/α_2C_ARKO mice (ARKOT). Representative images of immunoblots are shown below respective charts. Data are shown as percentage of WT control group values (set to 100%) and presented as mean ± standard error from mean. *p<0.05 vs. age-matched WT; #p<0.05 vs. age-matched ARKO.

In order to verify whether AET would affect skeletal UPS activation in our model, we measured protein ubiquitination, chymotrypsin-like proteasome activity and expression of proteasome subunits in WT, untrained and aerobic exercise-trained α_2A_/α_2C_ARKO mice. Ubiquitinated proteins expression was significantly increased in α_2A_/α_2C_ARKO mice and AET effectively reduced it to WT levels (Figure 4A). As mentioned above, proteasome activity was increased in 7 month-old α_2A_/α_2C_ARKO mice (Figure 4B) even though no changes were observed in proteasome subunits expression (data not shown). AET significantly reduced proteasomal activity, restoring the WT pattern (Figure 4B). Importantly, normal levels of protein carbonylation accompanied restoration of ubiquitinated protein levels and proteasome activity in trained mice (Figure 4).

### AET Effects on Skeletal Muscle Trophicity and Function in α2A/α2CARKO Mice

**Figure 5:**
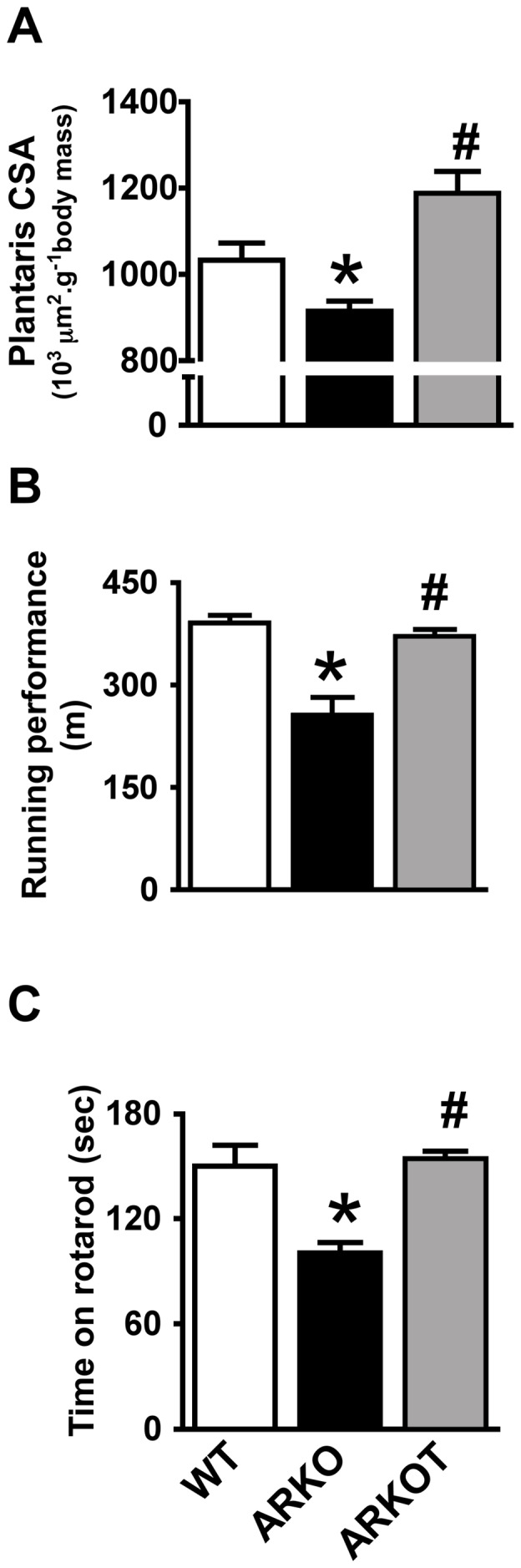
Skeletal muscle structure and global function . Plantaris cross-sectional area (CSA) (A), running performance (B) and time on rotarod (C) in 7 month-old wild type (WT), untrained α_2A_/α_2C_ARKO (ARKO) and trained α_2A_/α_2C_ARKO mice (ARKOT). Data are presented as mean ± standard error from mean. *p<0.05 vs. age-matched WT; #p<0.05 vs. age-matched ARKO.

To test whether afore-mentioned effects of AET on UPS components were associated with improved skeletal muscle phenotype, plantaris CSA, running capacity and rotarod performance were assessed. Plantaris atrophy and skeletal muscle global dysfunction were completely prevented by AET in our HF model (Figure 5), confirming improved skeletal muscle phenotype by the intervention. Representative histological images are shown in Information S4. Additionally, our group demonstrated in previous publications that AET was able to prevent exercise intolerance, metabolic impairment (e.g. maximal activity of citrate synthase and hexokinase), fiber type shift toward type II fibers and calcium handling disturbances on skeletal muscle in the same animal model used in the present investigation [21], [25].

### Physiological AET Effects in Human HF

HF patients submitted to AET showed increased peak oxygen uptake when compared with untrained HF patients (Table 3). Likewise, work economy at submaximal intensity was also improved in trained patients (9.4±0.7 vs. 7.3±0.4 mlO_2_.min^-1^.kg^-1^ in sedentary and trained HF patients, respectively, during exercise at a fixed submaximal intensity; p<0.05).

**Table 3:** Patient physiological parameters.

	Control	HF-S	HF-T
**Age, years**	72.2±1.6	75.0±6.5	75.0±5.5
**BMI, kg/cm^2^**	24.1±1.1	25.5±2.2	24.7±3.0
**VO_2_ peak, mLO_2_.kg^-1^.min^-1^**	37.0±2.9	12.4±0.5*	14.5±0.4* #
**RER at VO_2_ peak**	1.05±0.01	1.12±0.01	1.10±0.02
**ACE inhibitors**	0/6	5/5	6/6
**β-Blockers**	0/6	5/5	6/6
**Statins**	0/6	5/5	6/6

Age, body-mass index (BMI), peak oxygen uptake (VO_2_peak), respiratory exchange ratio (RER) at VO_2_peak and used medication in healthy individuals (Control), sedentary heart failure patients (HF-S) and trained heart failure patients (HF-T). Data are presented as mean ± standard error from mean.

* p<0.05 vs. Control.

# p<0.05 vs. HF-S.

### Skeletal Muscle Proteasome Activation in Human HF and Effects of AET

**Figure 6:**
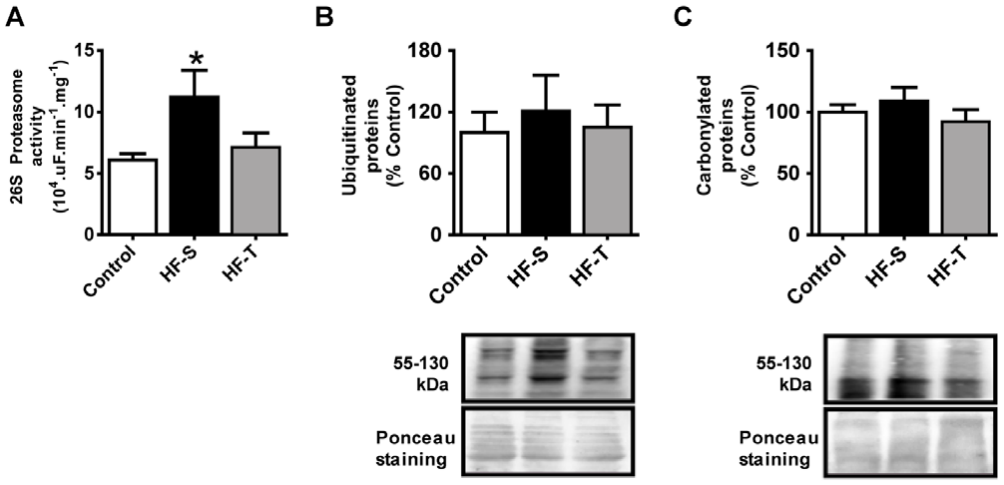
Skeletal muscle UPS in human HF. Chymotrypsin-like proteasome activity (A) and protein ubiquitination (B) and carbonylation (C) in healthy control subjects (Control), sedentary (HF-S) and trained HF (HF-T) patients. Immunobloting data are shown as percentage of Control group (set to 100%). Data are presented as mean ± standard error from mean. *p<0.05 vs. Control.

Similarly to observed in HF mice, chymotrypsin-like proteasome activity in skeletal muscle biopsies from HF patients was increased in comparison with healthy individuals (Figure 6A). Importantly, skeletal muscle from trained patients presented normal proteasome activity (Figure 6A). Protein ubiquitination and carbonylation were unchanged among groups (Figures 6B and C).

## Discussion

A wealth of data suggests AET as a key intervention for prevention and treatment in cardiology. Recent reports demonstrate protection provided by AET against HF-induced skeletal myopathy [20], [21], [25]. However, the molecular mechanisms by which AET delay or reverse skeletal muscle myopathy in HF remain elusive. Several key findings emerge from this study, in which we analyzed the contribution of AET in preventing plantaris atrophy in sympathetic hyperactivity induced-HF mice: (a) progressive skeletal muscle loss in α_2A_/α_2C_ARKO mice was associated with increasingly oxidative stress and proteasomal activity; (b) UPS overactivation and oxidative damage were detected when plantaris muscle atrophy was established in 7 month-old α_2A_/α_2C_ARKO mice; (c) AET efficiently reestablished plantaris phenotype, UPS and oxidative stress to WT levels. In human HF: (d) increased skeletal muscle proteasome activity suggests overactivation of UPS and (e) AET restored proteasome activity to healthy control levels.

Overwhelming evidence demonstrates a striking association between disease-induced skeletal muscle atrophy and UPS activation [4], which also occurs in HF [34], [35]. In fact, ubiquitination of skeletal muscle contractile proteins has been suggested in HF [36]. In line with these findings, we demonstrate that plantaris atrophy in HF mice is associated with UPS overactivation and that these phenomena display interesting relationship with severity of the disease, since skeletal myopathy was associated with worsening cardiac function and clinical signs of HF.

At 3 months of age, when α_2A_/α_2C_ARKO mice display preserved cardiac function and exercise tolerance, plantaris hypertrophy is observed in comparison with WT, which might be explained by β_2_-adrenoceptor overactivation due to sympathetic hyperactivity, as previously demonstrated [21]. Moreover, decreased proteasomal activity in 3 month-old α_2A_/α_2C_ARKO mice indicates reduced skeletal muscle proteolysis, favoring muscle hypertrophy. This finding might also be related to β_2_-adrenoceptor overactivation due to sympathetic hyperactivity, which activates hypertrophic signaling pathways besides inhibiting UPS activation in skeletal muscle in atrophic states [37], [38]. At 5 months of age, skeletal muscle hypertrophy was no longer observed and proteasomal activity was similar between α_2A_/α_2C_ARKO and WT. However, when severe HF was established in 7 month-old α_2A_/α_2C_ARKO mice, plantaris muscle atrophy and substantial proteasomal overactivation were detected. Among the possible mechanisms contributing to these observations, oxidative stress should be highlighted, since increasing lipid hydroperoxidation and protein carbonylation from 3 to 7 months of age were observed in α_2A_/α_2C_ARKO mice and redox unbalance is known to modulate UPS activation and leads to skeletal muscle atrophy.

Increased oxidative stress arises from imbalance between pro- and antioxidant activity [39] and is depicted in skeletal muscle under catabolic or dysfunctional states [6], [7], [40], [41]. Importantly, while increased oxidative stress through superoxide dismutase (SOD) deletion accelerates aging-induced skeletal muscle atrophy [40], antioxidant treatment effectively attenuates skeletal muscle loss in a cancer model [6]. Therefore, strong evidence of redox imbalance-induced skeletal muscle atrophy supports our hypothesis that oxidative damage is a major determinant of skeletal muscle loss in HF.

UPS modulation by redox balance depends upon oxidative damage extension. Protein damage by mild or moderate redox imbalance increases UPS substrate availability, causing elevation of proteasome activity [42]. Conversely, severe oxidative damage impairs substrate tagging by E3 ligases and causes proteasomal dysfunction due to accumulation of non-degradable aggregates [42], resulting in overall UPS inactivation. Therefore, we suggest that skeletal muscle oxidative stress reaches moderate levels in 7 month-old α_2A_/α_2C_ARKO mice, since accumulation of lipid hydroperoxides and carbonylated proteins were observed concomitantly with UPS overactivation.

Studies suggest proteasome inhibition as a treatment against skeletal muscle loss [43], [44]. However, potentially dangerous effects of such intervention must be considered, since the UPS is a major effector of the protein quality control mechanism in all cells [45], [46]. In fact, cardiac dysfunction occurs when the proteasome is inhibited *in vivo* [47]. In contrast, AET undoubtedly promotes beneficial effects in several tissues, including cardiac and skeletal muscles in HF [18], [48]. Thus, we have recently shown that AET prevents skeletal muscle atrophy in our experimental model [21], and here we extend our findings to the preventive effect of AET on oxidative stress and UPS overactivation.

Restoration of redox balance by AET is probably driven by antioxidant defense, such as augmented activity of free radical scavengers and reduced levels of inflammatory cytokines [49], [50]. Accordingly, we demonstrate here that AET reduced skeletal muscle lipid hydroperoxidation and protein carbonylation, accounting for reduced intracellular stress and relief of UPS overload. Therefore, reduced UPS activation by AET possibly occurred due to improvements in redox balance. These results suggest that AET ultimately counteracts increased protein degradation by the UPS.

Besides UPS overactivation by oxidative stress, it may also be purposed that redox unbalance is involved in HF-induced skeletal myopathy by reducing skeletal muscle regenerative capacity through disturbance of satellite cell pool or differentiation rate [51], [52]. Indeed, direct negative effects of oxidative stress on skeletal muscle satellite cells have been reported [53]. Furthermore, it has been shown that HF patients present depressed IGF-1 signaling in skeletal muscle [54] and that anabolic effects of IGF-1 are partially attributed to satellite cells activation [55], [56]. Following this rationale and considering that satellite cells activation is the leading process mediating muscle regeneration, it is also reasonable to speculate that anti-atrophic effects of AET could be blunted in our model, even though we observed several beneficial outcomes. In this sense, investigation of satellite cells participation in cardiac cachexia is a promising topic for future studies.

In human HF, AET also improved aerobic capacity (VO_2_peak) and work economy, which were mainly due to skeletal muscle improvements, since cardiac function did not differ between sedentary and trained HF patients (28±5 vs. 35±2% are EF values in sedentary and trained HF patients, respectively; p>0.05). Importantly, skeletal muscle UPS overactivation is suggested by increased proteasome activity in sedentary HF patients, corroborating findings of a recent study that presented increased abundance of MuRF1 in skeletal muscle in a larger population of HF patients, regardless of age [57]. This same study also showed that MuRF1 expression is reduced by AET, which goes in line with our finding that AET reverted 26 S Proteasome overactivation in HF patients.

Increased proteasomal activity was not accompanied by increased protein carbonylation, indicating that our HF patients displayed only mild skeletal muscle oxidative stress, but sufficient to induce myofibrillar protein damage and proteasomal activation. It is important to highlight that all HF patients were under β-blockade, ACE inhibition and statins, which independently provide antioxidant effects [58]-[60] and may have relieved skeletal muscle oxidative stress. However, we reinforce the role of AET in HF treatment by showing that even optimal drug treatment does not improve aerobic capacity and could not maintain skeletal muscle proteasomal activity, which is clearly achieved by AET.

### Study Limitations

Our study shows that AET reduced oxidative stress, UPS overactivation and prevented skeletal muscle atrophy in HF mice, however, it does not provide direct evidence of cause-effect among these findings. However, our hypothesis that relieved oxidative stress counteracts UPS overactivation is partly supported by the literature [42]. One might argue that our sympathetic hyperactivity-induced HF model is not the most similar to human HF, however, we provided strong evidence that the progression of HF in our model recapitulates many aspects of human HF [23]. Since enrolled HF patients were under optimal pharmacological therapy, we could not isolate the effects of AET. Additionally, small biopsy fragments didn’t allow further exploration of UPS modulation and evaluation of mild oxidative stress indicators, such as lipid hydroperoxidation. Left ventricular FS values in WT mice were lower than found in our previous work [23], which probably occurred due to the anesthetic agent used in the present study (halothane presently used vs. isoflurane previously used [23]). Even though halothane depresses cardiac contractility in a higher degree than isoflurane [61], we were able to reproduce the same pattern of cardiac dysfunction in our HF model and the effects of exercise training [21], [25], [28], [30], [31].

In conclusion, we provide evidence that AET prevents skeletal muscle oxidative stress and UPS activation in HF mice, which probably contributes to prevention of skeletal myopathy. The clinical relevance of the present investigation is demonstrated by attenuation in skeletal muscle proteasome activity in exercise-trained HF patients, which is not achieved by drug treatment itself. Altogether these findings strengthen AET as an efficient non-pharmacological tool for HF therapy.

## Supporting Information

Information S1 **CONSORT checklist for non-pharmacological trials.**
(DOC)

Information S2 **Primer sequence used for real-time PCR.**
(DOC)

Information S3 Cumulative survival of wild type (WT), untrained α_2A_/α_2C_ARKO (ARKO) and trained α_2A_/α_2C_ARKO mice (ARKOT) after starting experimental protocol, when mice were 5 month-old. ***p = 0.002 vs. WT.
(DOC)

Information S4 **Representative histological images of untrained wild type (WT) and α_2A_/α_2C_ARKO (ARKO) mice at 3, 5 and 7 months of age and trained α_2A_/α_2C_ARKO mice (ARKOT) at 7 months of age.** Muscle sections were prepared and analyzed as described in the Methods section of the manuscript. Dashed lines represent the location of plantaris muscle. Same magnification (50x) was applied to all images.
(DOC)
