# Supplementary material for: Exercise Training Prevents Oxidative Stress and Ubiquitin-Proteasome System Overactivity and Reverse Skeletal Muscle Atrophy in Heart Failure
Source: PLoS One. 2012 Aug 3;7(8):e41701. doi: 10.1371/journal.pone.0041701 (PMC3411696; doi:10.1371/journal.pone.0041701)
Supplement: Information S2 — Primer sequence used for real-time PCR. (DOC) [file pone.0041701.s002.doc]

**Supplementary Information S2**

***Primer sequence used for Real Time PCR:***

| Atrogin-1 |
| --- |
| sense: 5’-TACTAAGGAGCGCCATGGATACT-3’ |
| anti-sense: 5’-GTTGAATCTTCTGGAATCCAG GAT-3’ |
|  |
| MuRF-1 |
| sense: 5’-GTGTGAGGTGCCTACTTGCT-3’ |
| anti-sense: 5’-ACTCAGCTCCTCCTTCACCT-3’ |
|  |
| E3-α |
| sense: 5’-ACGTGTGTGCTCTGTATGGA-3’ |
| anti-sense: 5’-GCTTGAGCAATCACCTCTTC-3’ |
|  |
| USP14 |
| sense: 5’CCTTTTCATTTGCTGACGATATTGGCTCC-3’ |
| anti-sense: 5’-CATAAGCAATGTGCCAGTCTCCACCACC-3’ |
|  |
| USP19 |
| sense: 5’-GTATCTCGGCCTGAAGCTGCTGTGC-3’ |
| anti-sense: 5’-CCAATGGTGTCTCCCCTTTGTCCTCC-3’ |
|  |
| USP28 |
| sense: 5’-AAAGGCCAGTAA TGGTGACATCA-3’ |
| anti-sense: 5’-GTCGTGACTGGGCTCCTTAACT-3’ |
|  |
| Cyclofilin |
| sense: 5’-AATGCTGGACCAAACACAAA-3’ |
| anti-sense: 5’-CCTTCTTTCACCTTCCCAAA-3’ |
