# Supplementary material for: Exercise Training Prevents Oxidative Stress and Ubiquitin-Proteasome System Overactivity and Reverse Skeletal Muscle Atrophy in Heart Failure
Source: PLoS One. 2012 Aug 3;7(8):e41701. doi: 10.1371/journal.pone.0041701 (PMC3411696; doi:10.1371/journal.pone.0041701)
Supplement: Information S3 — Cumulative survival of wild type (WT), untrained α2A/α2CARKO (ARKO) and trained α2A/α2CARKO mice (ARKOT) after starting experimental protocol, when mice were 5 month-old. ***p = 0.002 vs. WT. (DOC) [file pone.0041701.s003.doc]

**Supporting information S3**

**
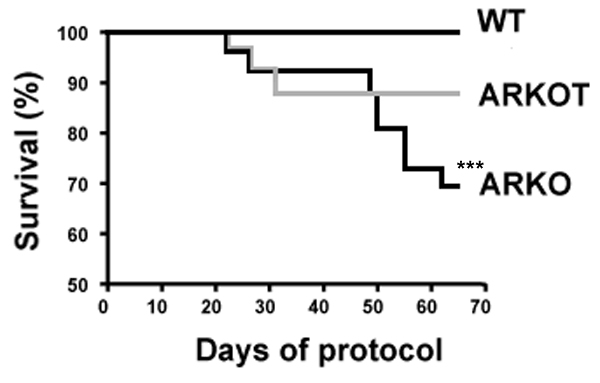
**

***Supporting information S3.*** Cumulative survival of wild type (WT), untrained α_2A_/α_2C_ARKO (ARKO) and trained α_2A_/α_2C_ARKO mice (ARKOT) after starting experimental protocol, when mice were 5 month-old. ***p=0.002 vs. WT.
