# Supplementary material for: Exercise Training Prevents Oxidative Stress and Ubiquitin-Proteasome System Overactivity and Reverse Skeletal Muscle Atrophy in Heart Failure
Source: PLoS One. 2012 Aug 3;7(8):e41701. doi: 10.1371/journal.pone.0041701 (PMC3411696; doi:10.1371/journal.pone.0041701)
Supplement: Information S4 — Representative histological images of untrained wild type (WT) and α2A/α2CARKO (ARKO) mice at 3, 5 and 7 months of age and trained α2A/α2CARKO mice (ARKOT) at 7 months of age. Muscle sections were prepared and analyzed as described in the Methods section of the manuscript. Dashed lines represent the location of plantaris muscle. Same magnification (50x) was applied to all images. (DOC) [file pone.0041701.s004.doc]

**Supporting Information S4**


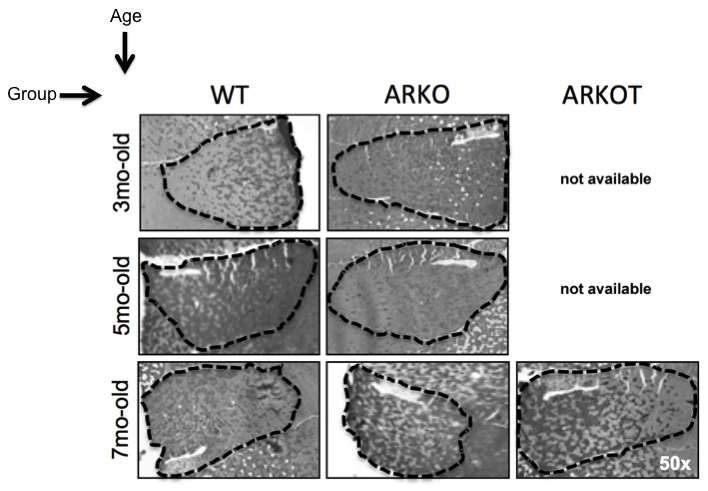


***Supporting Information S4.*** Representative histological images of untrained wild type (WT) and α_2A_/α_2C_ARKO (ARKO) mice at 3, 5 and 7 months of age and trained α_2A_/α_2C_ARKO mice (ARKOT) at 7 months of age. Muscle sections were prepared and analyzed as described in the Methods section of the manuscript. Dashed lines represent the location of plantaris muscle. Same magnification (50x) was applied to all images.
